# Same sensitivity with shorter exposure: behavior as an appropriate parameter to assess metal toxicity

**DOI:** 10.1007/s10646-022-02584-w

**Published:** 2022-09-16

**Authors:** Álvaro Alonso, Alberto Romero-Blanco

**Affiliations:** Universidad de Alcalá, Facultad de Ciencias, Departamento de Ciencias de la Vida, Unidad de Ecología, Research Group in Biological Invasions, Campus Científico Tecnológico, Alcalá de Henares, 28805 Madrid Spain

**Keywords:** Metal toxicity, Aquatic animals, Ecotoxicological bioassays, Chemical toxicity distributions

## Abstract

The exposure of animals to toxicants may cause a depletion in the energy uptake, which compromises reproduction and growth. Although both parameters are ecologically relevant, they usually need long-term bioassays. This is a handicap for the availability of toxicological data for environmental risk assessment. Short-term bioassays conducted with environmental concentrations, and using relevant ecological parameters sensitive to short-term exposures, such as behavior, could be a good alternative. Therefore, to include this parameter in the risk assessment procedures, it is relevant the comparison of its sensitivity with that of growth and reproduction bioassays. The study aim was the assessment of differences between endpoints based on mortality, behaviour, reproduction, and growth for the toxicity of metals on aquatic animals. We used the ECOTOX database to gather data to construct chemical toxicity distribution (CTD) curves. The mean concentrations, the mean exposure time, and the ratio between the mean concentration and the exposure time were compared among endpoints. Our results showed that behavioral, growth, and reproduction bioassays presented similar sensitivity. The shortest exposure was found in behavioral and reproduction bioassays. In general, the amount of toxicant used per time was lower in growth and reproduction bioassays than in behavioral and mortality bioassays. We can conclude that, for metal toxicity, behavioral bioassays are less time-consuming than growth bioassays. As the sensitivity of behavior was similar to that of growth and reproduction, this endpoint could be a better alternative to longer bioassays.

## Introduction

Different studies at field and laboratory scales have been developed to assess the deleterious effects of chemicals on ecosystems (Chapman, 2002; Coulaud et al., 2015; Hanson et al., 2017; Peterson et al., 2017; Ali et al., 2019). Mortality is usually monitored in laboratory bioassays conducted at short-term exposures for different animal species, including invertebrates and fish. However, lethality is caused at very high concentrations and at very short exposure time, which is not usually representative of polluted ecosystems (Scott and Sloman, 2004; Robinson, 2009; Peterson et al., 2017). These bioassays present unquestionable advantages, as they are not time-consuming (Melvin and Wilson, 2013; Alonso et al., 2016). This is an important issue, as the chemical industry releases hundreds of novels chemicals into the environment every year, which makes short-term lethal bioassays the base for most ecotoxicological studies (Vitousek et al., 1997; Newman, 2015). Regulatory decisions are partly based on data from the peer-reviewed literature, which makes lethal short-term bioassays the main source of the decision-making (Solomon et al., 2002; Scott and Sloman 2004; Hanson et al. 2017). However, animals are usually exposed to low chemical concentrations during chronic or pulse exposures in natural ecosystems (Ashauer et al., 2007; Trac et al., 2016; Peterson et al., 2017). That exposure may cause a depletion in the energy uptake, which compromises the reproduction and growth. Both parameters are ecologically relevant, but long-term bioassays are often needed to assess the effects of depletion of energy uptake, especially in species with long life cycles, which is a handicap for their broad use in ecotoxicology and fast decision making (González-Doncel et al., 2006; Gerhardt, 2007; Hellou, 2011; Melvin and Wilson, 2013). Therefore, the availability of growth and reproduction data in ecotoxicological database is scarce in comparison with studies focus on lethality.

The environmental risk assessment (ERA) is a procedure for making decisions based on the risk of chemicals to natural ecosystems (Solomon et al., 2002; Zweers and Vermeire, 2007). The ERA for a toxicant is conducted with the available ecotoxicological information, which comes mostly from bioassays on short-term effects for different species (Traunspurger and Drews, 1996; Solomon et al., 2002; Zweers and Vermeire, 2007; Rodrigues et al., 2017). To refine this procedure, data of growth and reproduction studies are also incorporated as well as studies of higher levels of organization (i.e., trophic, microcosm, mesocosm, or ecosystem studies) by means of a tiered approach (Zweers and Vermeire, 2007; Daam et al., 2013). These studies are more realistic, as they use longer exposure time and lower toxicants concentrations. Unfortunately, there is a gap of information on the basic toxicity for most of the manufactured compounds (Zweers and Vermeire, 2007; Williams et al., 2011). The main reasons are the high rate of new chemicals production and the cost of performing new ecotoxicological bioassays (Vitousek et al., 1997; Denoël et al., 2013; Melvin and Wilson, 2013; Newman, 2015; Alonso et al., 2016). A trade-off may be the development of bioassays that can be conducted in a relatively short period at realistic environmental concentrations and with relevant ecological parameters (Gerhardt, 2007; Hellou, 2011; Denoël et al., 2013; Melvin and Wilson, 2013; Alonso et al., 2016). Behavioral parameters may facilitate the achievement of this task, as (1) it has been demonstrated that behavioral bioassays are conducted at shorter exposures than growth and reproduction bioassays (Melvin and Wilson 2013), (2) behavior has been considered as an early warning tool (Hellou, 2011), and (3) behavior is among the most sensitive parameters of toxicant exposure at realistic environmental concentrations (Traunspurger and Drews, 1996; Gerhardt, 2007; Hellou, 2011). Behavior is an essential parameter to understand the fitness of individuals and population, and it has also been suggested as a good index of sublethal toxicity for wildlife (Warner et al. 1966; Little 1990; Traunspurger and Drews 1996; Bryan et al. 1995). Despite these advantages, the inclusion of behavior in ecotoxicological risk assessment is still scarce.

For the development of new bioassays, it is relevant to compare among different endpoints to understand their handicaps and advantages. Distribution models (such as species distribution or chemical toxicity distribution) are an appropriate way to assess the effects of toxicants on a group of organisms or endpoints (González-Doncel et al., 2006; Williams et al., 2011), since they allow the use of a broad sets of ecotoxicological data to test hypotheses (González-Doncel et al., 2006; Dobbins et al., 2008; Williams et al., 2011; Liu et al., 2014).

Among toxicants, metals represent a threat to aquatic organisms, as they are highly toxic even at low concentrations (Newman, 2015; Ali et al., 2019; Gradinaru et al., 2019). Metals have been amply studied at different scales, aquatic environments, and taxonomic groups (Bryan et al., 1995; Mayer-Pinto et al., 2010; Newman, 2015; Gissi et al., 2016; Ali et al., 2019; Gradinaru et al. 2019; Sadeq and Beckerman, 2019). Additionally, their toxicological effects have been studied at individual scale, including mortality, behavior, reproduction, and growth endpoints (Alonso et al., 2009, 2010; Martins et al., 2017; Ali et al., 2019; Sadeq and Beckerman, 2019; Wang et al., 2020). Therefore, metals are good candidates to compare the advantages and disadvantages of mortality, growth, reproduction, and behavior endpoints.

The aim of this study is to assess the differences between endpoints based on mortality, growth, reproduction, and behavior for three variables: exposure time, toxicant concentration, and the rate between toxicant concentration and exposure time. For that purpose, available data on metal-metalloid toxicity to aquatic animals and protozoa have been gathered to build chemical toxicity distribution curves. We hypothesized that endpoints based on behavior will represent a faster parameter than growth and reproduction, and they will be relatively more sensitive endpoints.

## Material and Methods

### Data selection

**Fig. 1:**
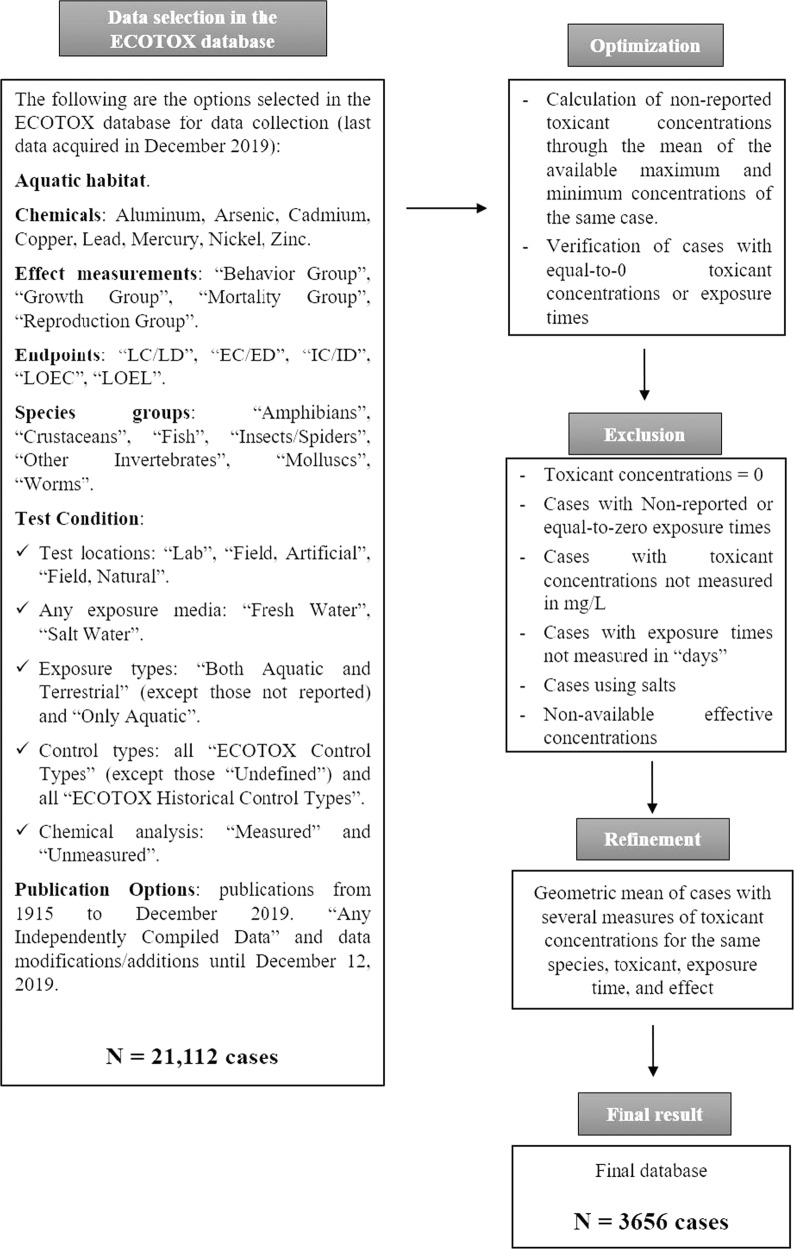
Flux diagram showing the data selected from the ECOTOX database and the subsequent optimization, exclusion, and refinement criteria

We used the US Environmental Protection Agency (US-EPA) ECOTOX database to collect the ecotoxicological data of the toxicity of metals-metalloids on aquatic animals and protozoa (https://cfpub.epa.gov/ecotox/, last data acquired in December 2019) (Fig. 1). The selected metals-metalloids were aluminium, arsenic, cadmium, copper, lead, mercury, nickel, and zinc (Fig. 1). They were chosen because their toxicity is supported by an extensive background of data (Newman, 2015). The target organisms were animal species with a whole or partial aquatic life cycle; amphibians, crustaceans, fish, aquatic insects, molluscs, aquatic worms, and other invertebrates (rotifers, bryozoa, sponges, cnidarians, and echinoderms) and protozoa (amoebas and ciliates) (Fig. 1). We selected several variables at individual level in **four effect groups** (Mortality, Behavior, Reproduction, and Growth) (Fig. 1). Behavioral variables were related to avoidance, movements and feeding. Reproduction variables related to progeny counts, egg size, spawning frequency, time to first progeny, egg fertilization and sperm properties. For growth, most of the variables were related to weight, length, growth rate, and condition index. Based on those variables, we selected **eight groups of endpoints**: Lethal Concentrations (LCs), Lethal Doses (LDs), Effective Concentrations (ECs), Effective Doses (EDs), Inhibition Concentrations (ICs), Inhibition Doses (IDs), Lowest Observed Effect Concentrations (LOEC), and Lowest Observed Effect Levels (LOEL) (Fig. 1). The names of endpoints match the terminology used in the ECOTOX database. All available studies with data from laboratory or field bioassays were gathered (Fig. 1). All reported exposure types, data that included aquatic media (both marine and freshwater), toxicant exposure in the aquatic media, control treatments, and nominal or/and actual toxicant concentrations were included. Data from non-reported or undefined categories were checked in the source reference and discarded if not available. All publications reported in ECOTOX that met these conditions were selected from 1915 until December 2019 (a total of 21,112 cases, Fig. 1).

Bioassays that used salts with other elements-compounds with likely toxicity (apart from the metal-metalloid toxicity) were rejected. If an entry did not report a mean toxicant concentration it was replaced -if available- by the mean of the reported maximum and minimum toxicant concentrations. Cases with toxicant concentrations and exposure times equal to 0 were also omitted after checking them in the respective studies. To avoid data repetition, we performed the geometric mean for those cases that reported several measures of toxicant concentrations for the same species, toxicant, exposure time, and effect. Therefore, a total of 3,656 cases were retained. Each case evaluates the toxicological effect of exposure to single metal-metalloid compounds or elements.

### Data analysis

For each effect group (mortality, behaviour, reproduction, and growth) and using all gathered endpoints for metals-metalloids and species, three Chemical Toxicity Distributions (CTD) were constructed (Gonzalez-Doncel et al., 2006; Williams et al., 2011). In these models, the logarithmic concentrations of all studied toxicants are situated along the X axis and the potentially affected fraction of species (i.e., percentage of species that are adversely affected with the increase of toxicant concentration) is displayed in the Y axis. The CTD for each effect group was constructed using the logarithm of the mean concentrations of the endpoints, the logarithm of the mean exposure times of the endpoints, and the logarithm of the ratio between the mean concentrations and exposure times in the X axis. This approach allows the assessment of the sensitivity, the bioassay duration, and the toxicant concentration used per exposure time in each effect group. Outliers were evaluated following the method of Jesenska et al. (2013), so that those endpoints outside of the 3σ (standard deviation) interval were considered as outliers. They were not included in analyses after checking their reliability in the original sources. Only one value had to be removed.

Lognorm or Weibull distributions were used for the fitting of the CTD distributions following by the Akaike’s, Bayesian, and Kolmogorov-Smirnov Goodness-of-Fit criteria. For each CTD curve, the 5 and 50 percentage Screening Point Value (SPV) were inferred (Williams et al., 2011). These values are the concentration or time or ratio concentration/exposure time that affects the 5 or 50 percentage of the studied cases for each model. The SPV for concentration (SPVC_5_ for 5% and SPVC_50_ for 50%) were inferred from concentration curves, SPV for time (SPVT_5_ and SPVT_50_) were inferred from exposure time curves, and the ratio Concentration/Time (CT_5_ and CT_50_) for the ratio concentration/exposure time curves. The variability of these values was assessed through parametric and non-parametric (for mortality) bootstrapping (1000 repetitions). Differences between SPVC, SPVT, and CT values were conducted through ratio tests with the R package “ecotox” (Hlina et al., 2021). This function is based on the method developed by Wheeler et al. (2006), which is an alternative to the method of comparing intervals to determine differences. According to this method, we first performed a generalized linear model (glm) with the affected fraction of species and concentrations. With the resulting outputs, we could assess differences through ratio tests (Wheeler et al., 2006). Statistics were conducted with the R software (R Core Team, 2020).

## Results

**Fig. 2:**
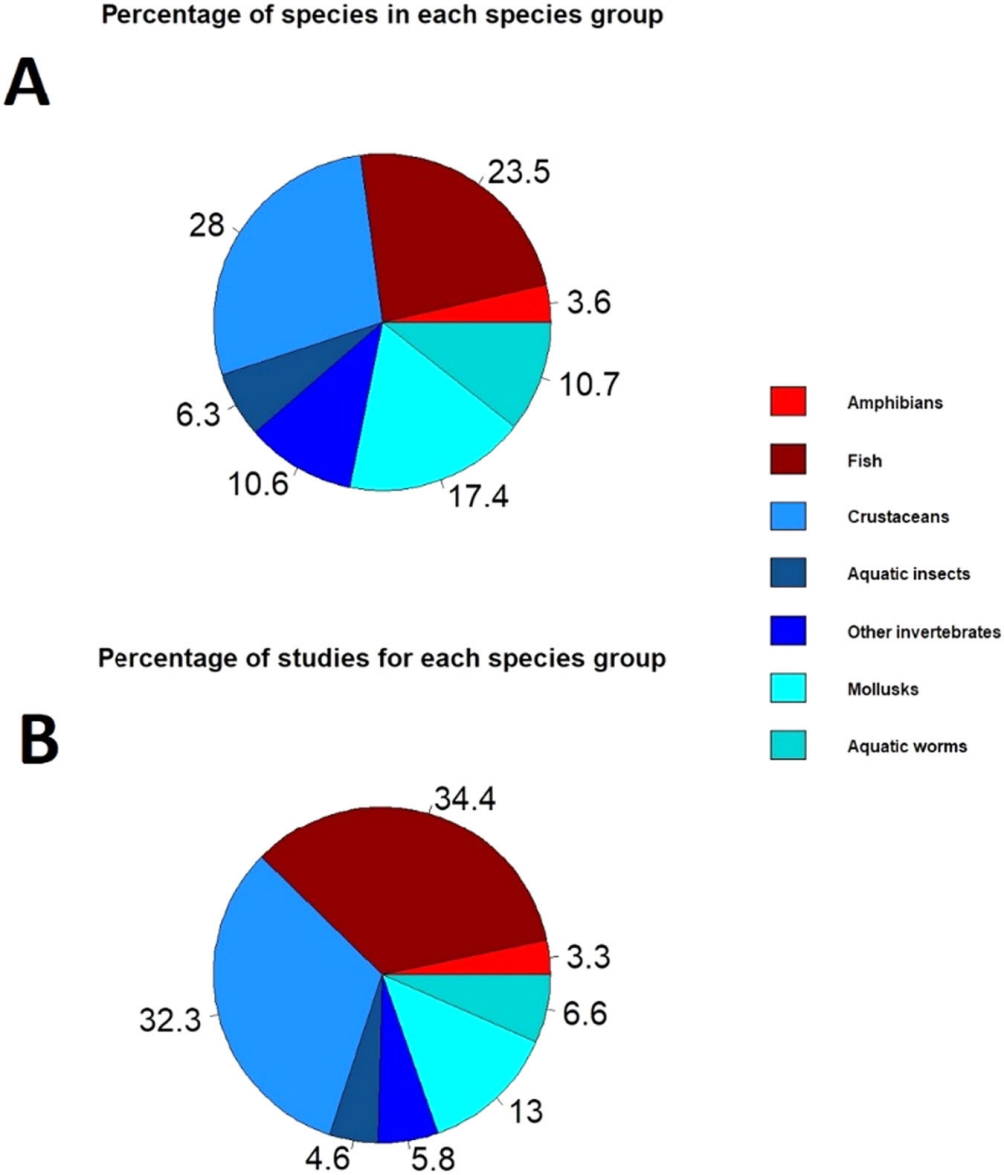
Percentage of species (**A**) and studies (**B**) in each animal group used for the Chemical Toxicity Distribution models

**Fig. 3:**
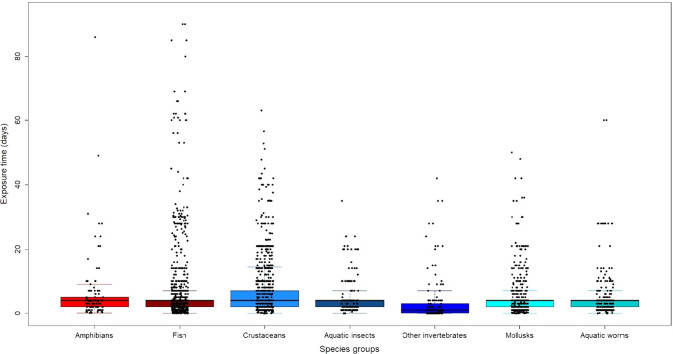
Mean exposure time to toxicants for each animal group used for the Chemical Toxicity Distribution (CTD) models. Black solid lines inside the boxes indicate the median. Filled black points represent the distribution of exposure time values

The total number of species for the models was 729 and the total number of studies from which data were obtained was 1,318. The groups with the highest number of species used for the Chemical Toxicity Distribution (CTD) models corresponded to crustaceans (204 species, 28% of the total number of species) and fish (171 species, 23.5% of the total number of species) (Fig. 2A). The highest number of studies was for the fish group (453) following by the crustacean group (425) (Fig. 2B). The mean duration of toxicant exposure for the studies in each animal group is presented in Fig. 3. The fish group showed a higher number of studies with longer exposures than other animal groups (Fig. 3). Most of the gathered endpoints for the models were Lethal Concentrations (LC) (2,916 cases), Effective Concentrations (EC) (312) and Lowest Observed Effect Concentrations (LOEC) (304) (Table 1). The compounds with the highest number of cases were cadmium (1598) and copper (854), while lead showed the lowest number of cases (61) (Table 2). Copper and cadmium also showed the cases with the longest exposure periods (Table 2).

**Table 1:** Number of cases and time ranks for each endpoint included in the study

Endpoints	Number of cases	Time ranks (days)
EC	312	<1–85
IC	34	<1–56
LC	2916	<1–100
LD	10	1–6
LOEC	304	<1–730.56
LOEL	56	<1–42

**Table 2:** Number of cases and time exposure ranks for each metal-metalloid

Metal group	Number of cases	Time ranks (days)
Aluminum	68	0.17–33
Arsenic	91	0.15–30
Cadmium	1598	1e−04–578.4
Copper	854	2e−04–730.6
Lead	61	0.67–42
Mercury	449	0.01–40
Nickel	138	0.08–85
Selenium	85	0.13–365.3
Zinc	312	0.01–139

**Fig. 4:**
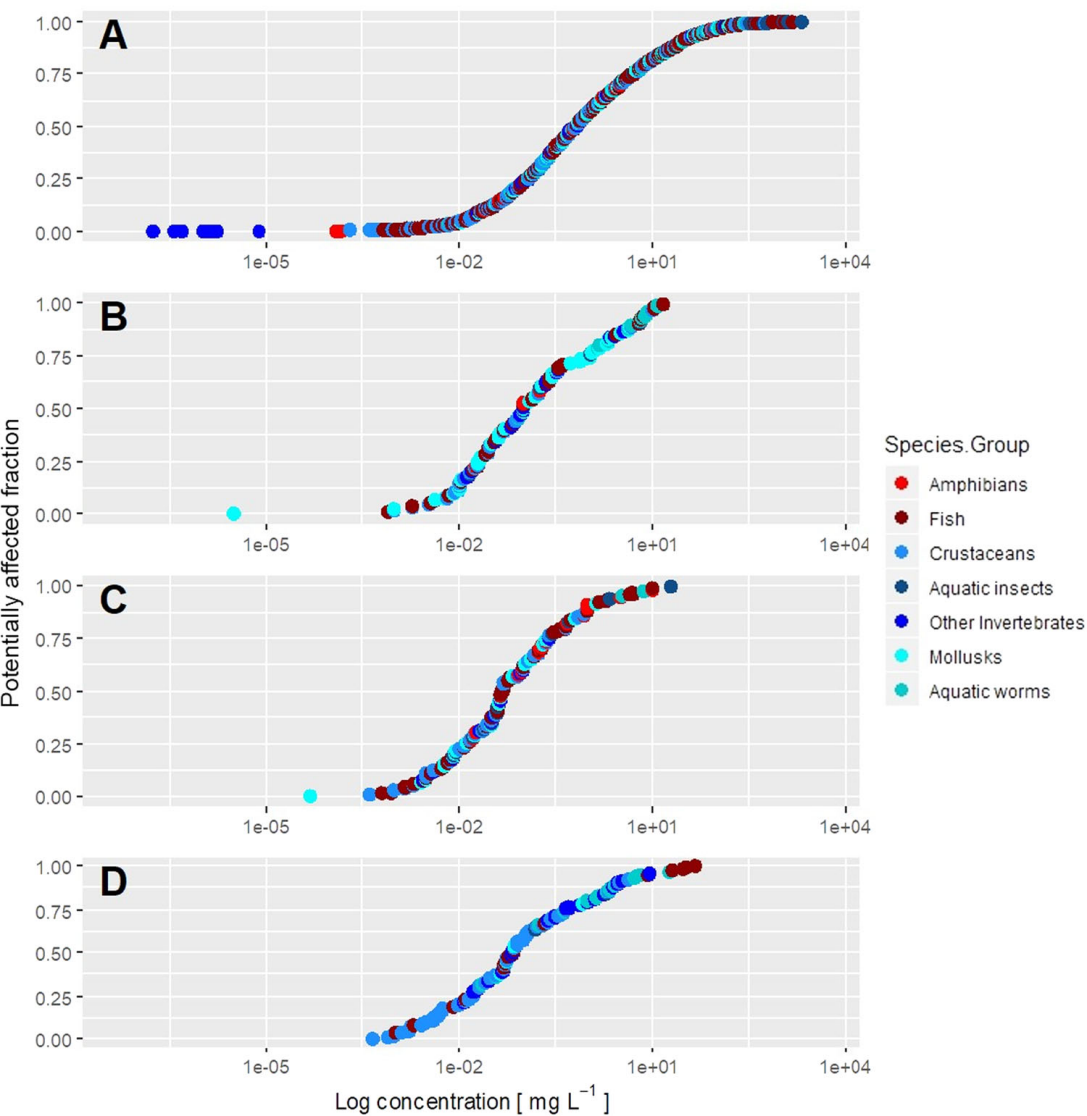
Representation of the Chemical Toxicity Distributions (CTDs) (X-axis was log-transformed for the toxicant concentration) with the cumulative distributions of the different species compiled from the ECOTOX database. Panels **A**, **B**, **C**, and **D** represent mortality, behavioral, growth, and reproduction endpoints, respectively

**Fig. 5:**
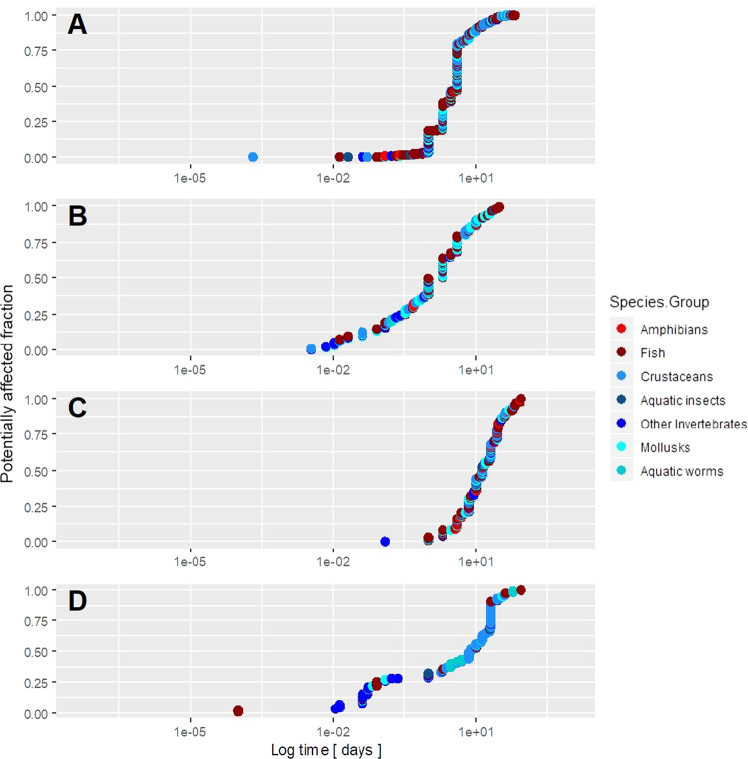
Representation of the Chemical Toxicity Distributions (CTDs) (X-axis was log-transformed for the exposure time) with the cumulative distributions of the different species compiled from the ECOTOX database. Panels **A**, **B**, **C**, and **D** represent mortality, behavioral, growth, and reproduction endpoints, respectively

**Fig. 6:**
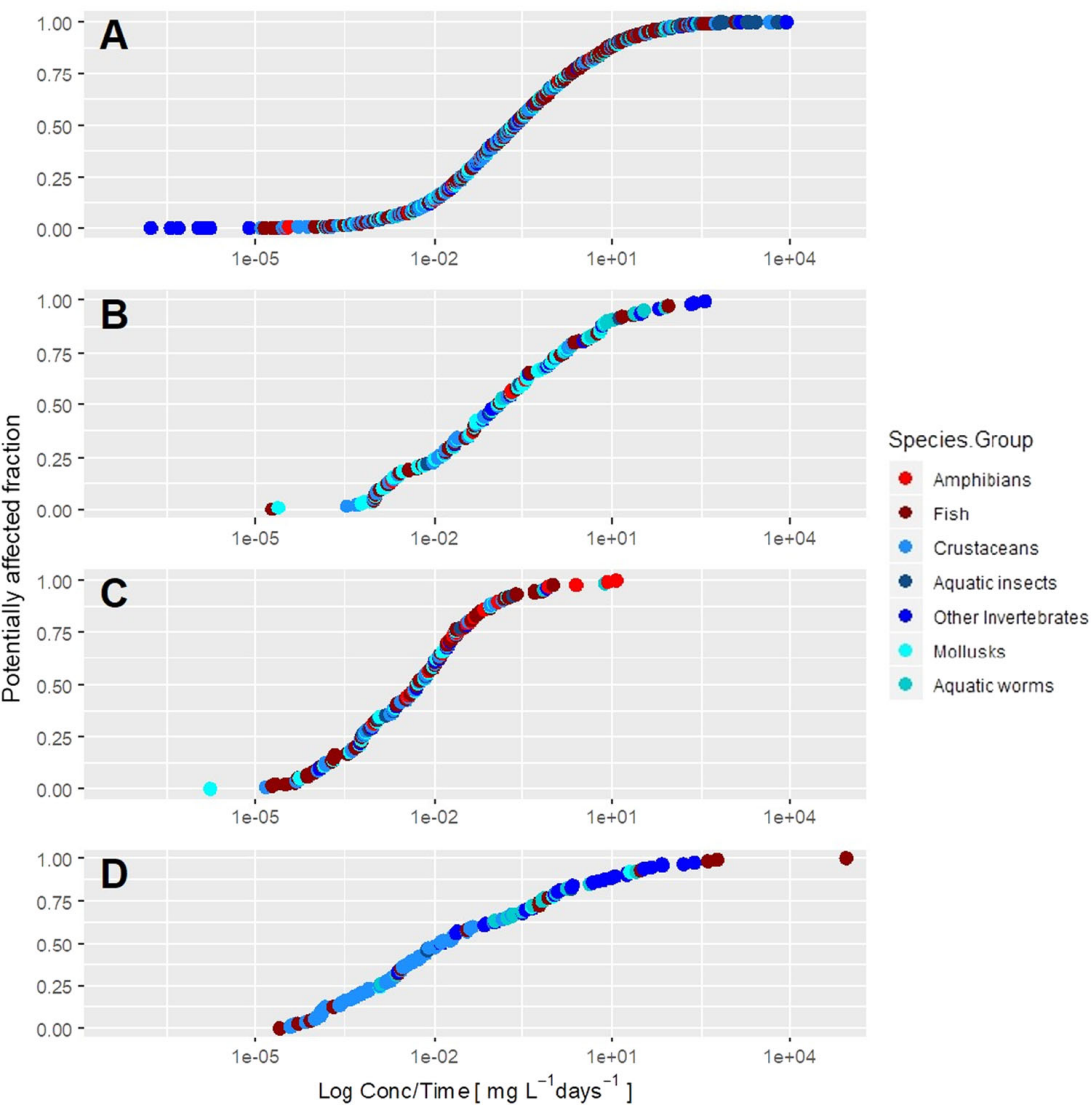
Representation of the Chemical Toxicity Distributions (CTDs) (X-axis was log-transformed for the ratio toxicant concentration/exposure time) with the cumulative distributions of the different species compiled from the ECOTOX database. Panels **A**, **B**, **C**, and **D** represent mortality, behavioral, growth, and reproduction endpoints, respectively

In general, the model for toxicant concentration based on mortality endpoints (Fig. 4A) showed a lower sensitivity than models based on behavior, growth, and reproduction (Fig. 4B, C, D, respectively). In the case of the exposure time, growth and reproduction models showed longer exposures than those of mortality and behavior (Fig. 5). In the case of the ratio of concentration per time unit, the highest values were observed in studies focused on mortality and behavior (Fig. 6).

The Screening Point Values for concentration (SPVC), time (SPVT), and the Concentration/Time ratio (CT) for 5 and 50 percentages are presented in Table 3. Behavioral, growth, and reproduction bioassays showed a similar sensitivity for the SPVC_5_ (ratio test, *p* > 0.05), with values ranging from 0.00067 to 0.0036 mg/l. SPVC_5_ for mortality showed the highest concentrations (ratio test, *p* < 0.05). For time (SPVT_5_), the shortest bioassays were those based on behavior and reproduction, and those with the longest duration were growth bioassays (ratio test, *p* < 0.05). Values for mortality showed an intermedia time between both groups, behavioral and reproduction, and growth bioassays (Table 3). In general, the amount of toxicant per time unit (CT_5_) were lower in growth and reproduction bioassays than in behavioral and mortality bioassays (ratio test, *p* < 0.05) (Table 3). A similar trend was observed for the same parameters at 50 percentage of measured effect, with a lowest amount of toxicant per time unit for growth bioassay and with the highest values for behavior and mortality bioassays (ratio test, *p* < 0.05) (Table 3).

**Table 3:** Screening Point Values for concentration (SPVC), time (SPVT), and Concentration/Time ratios (CT) for 5 and 50 percentages of the studied cases are presented for each effect (Behavior, Mortality, Growth, and Reproduction). For each value, the respective 95% confidence interval is presented. Different letters in confidence intervals show significant differences among values (ratio test). Asterisks in the Effect column mean that the Weibull fitting method was applied. Otherwise, the lognorm fitting method was used

Screening Point Values	Effect	Value	Bootstrap (two-sided 95%)
SPVC_5_ (mg/l)	Behavior	0.0019	0.0011–0.0036^a^
	Mortality	0.0075	0.0062–0.0089^b^
	Growth	0.0014	0.00088–0.0025^a^
	Reproduction	0.0013	0.00067–0.0025^a^
SPVT_5_ (days)	Behavior*	0.012	0.0064–0.030^a^
	Mortality	0.63	0.58–0.69^b^
	Growth	2.18	1.70–2.80^c^
	Reproduction	0.013	0.0064–0.028^a^
CT_5_ (mg/l/days)	Behavior	0.00048	0.00022–0.0011^b^
	Mortality	0.0013	0.0010–0.0015^b^
	Growth	4.84e−05	2.64e−05–9.07e−05^a^
	Reproduction	2.35e−05	7.88e−06-7.37e−05^a^
SPVC_50_ (mg/l)	Behavior	0.13	0.085–0.19^a^
	Mortality	0.72	0.65–0.80^b^
	Growth	0.059	0.043–0.085^a^
	Reproduction	0.088	0.058–0.14^a^
SPVT_50_ (days)	Behavior*	1.73	1.10–2.14^a^
	Mortality	3.13	3.02–3.23^b^
	Growth	12.70	10.80–14.74^c^
	Reproduction	2.00	1.20–3.43^a,b^
CT_50_ (mg/l/days)	Behavior	0.13	0.073–0.21^c^
	Mortality	0.23	0.21–0.26^c^
	Growth	0.0046	0.0030–0.0069^a^
	Reproduction	0.030	0.014–0.062^b^

## Discussion

We found the highest values of SPVC_5_ and SPVC_50_ for bioassays based on mortality, which means that these bioassays are less sensitive than those based on behavioral, growth, and reproduction endpoints. Additionally, mortality, behavioral, and reproduction bioassays are less time-consuming than growth studies. Growth and reproduction bioassays used less toxicant concentration per exposure time than behavioral and mortality bioassays. Latter show that they are similar with respect to the amount of toxicant used per unit of time. Nevertheless, the lower values of SPVCs confirm the greater sensitivity of behavioral parameters with respect to lethality.

Most of the toxicity testing in ecotoxicology focuses on the assessment of the direct effects of toxicants on organisms. To increase the realism of these bioassays, the use of environmental concentrations (i.e., low toxicant concentrations) and endpoints with ecological significance (i.e., endpoints that are linked with higher organization levels) are two essential requirements (Hellou, 2011; Denoël et al., 2013; Melvin and Wilson, 2013). Growth and reproduction endpoints allow the testing of low toxicant concentrations on two essential parameters, but they usually need long-term exposures (Melvin and Wilson, 2013). In our study, the Screening Point Values for Concentration (SPVC) showed similar values among behavior, growth, and reproduction. Therefore, the three effects seem useful when testing low concentrations of metals. This is a great advantage since the high rate of production of new compounds causes the pressing necessity to generate new ecotoxicological data to conduct a proper risk assessment (Zweers and Vermeire, 2007; Williams et al., 2011; Melvin and Wilson, 2013; Newman, 2015; Alonso et al., 2016). In this context, long-term bioassays may be a handicap to achieve that aim (Denoël et al. 2013). Therefore, the use of bioassays based on endpoints with shorter duration and higher or similar sensitivity should be a priority in ecotoxicology. This would allow the assessment of sublethal effects of more chemicals in shorter time (Denoël et al., 2013). Melvin and Wilson (2013) showed through a meta-analysis that behavioral bioassays are conducted at shorter exposures than reproduction and growth bioassays for a total of 52 aquatic species. Behavioral endpoints are generally more sensitive and less time-consuming than growth and reproduction endpoints for several compounds, including pesticides, metals, and pharmaceuticals among other (Melvin and Wilson, 2013). The same authors showed contrasting results for the sensitivity among bioassays based on behavior, reproduction, and growth for crustaceans and fish. Behavior has been reported as an alternative endpoint to assess the adverse effects of toxicants on animals, being regarded as one of the most sensitive endpoints of chemical stress (Dell’Omo, 2002).

In our study (focused on metals-metalloids) the sensitivity of behavior was very similar to that for growth and reproduction, the exposure time being shorter in behavioral bioassays than in growth studies. This result is not fully in concordance with Melvin and Wilson (2013) as these authors showed that for zinc and mercury behavior did not show the highest sensitivity. Although the difference in methodology makes difficult the direct comparison of both studies. The meta-analysis of Melvin and Wilson (2013) and the present study agree that behavior is a less-time consuming endpoint. In fact, there is scientific evidence of the links between behavioral impairments and whole organism responses, such as survival, reproduction, and growth (Martinović et al., 2007; Hellou, 2011; Denoël et al., 2013). This scientific evidence has been shown for several animal groups and toxicants, such as amphibians, fish, and invertebrates (Martinović et al., 2007; Hellou, 2011; Agatz et al., 2013; Denoël et al., 2013).

Previous studies have shown that behavior is a useful endpoint to assess the adverse effects of different toxicants on several animal species (Dell’Omo, 2002; Hellou, 2011; Denoël et al., 2013; Melvin and Wilson, 2013; Alonso et al., 2016). Among the different tested behaviors, those focused on feeding and food acquisition (i.e., all movements and activities that are associated with locating and ingesting food) have proven to be very sensitive parameters (Pyatt et al., 2002; Alonso et al., 2009, 2016; Alonso and Valle-Torres 2018). In our study several tested behaviors are related to feeding, including filtration rate, smell, valve closure, fecal production, feeding behavior, and food consumption. The rest of tested behaviors are mainly related to animal movements (such as swimming, ability to detach from substrate, or chemical avoidance).

Several metals cause an alteration in feeding behavior that subsequently reduce the growth of animals. For instance, cadmium caused a significant reduction in food consumption of the freshwater snail *Lymnaea luteola* and the subsequent deleterious effect on its growth rate (Das and Khangarot, 2010). In *L. stagnalis*, lead affected the feeding behavior and their ability to reach the food (Pyatt et al., 2002). Woodward et al., (1994) showed that a contaminated-metals diet inhibited the feeding activity of the fish *Salmo gairdneri*. The deleterious effects on the feeding behavior suppose an early warning in the feeding depletion, which may suppose a reduction in survival, growth, and reproduction in toxicant-exposed aquatic organisms. Those parameters are closely related with population dynamics (Jensen et al. 2001; Alonso and Valle-Torres 2018), so behavior could be a promising link with higher ecological organization levels. Therefore, feeding behavior studies could be a good alternative to long-term growth and reproduction bioassays (Alonso and Valle-Torres, 2018).

Our review has shown that most ecotoxicological studies with metals are carried out with fish and crustacean groups (66.7%), while amphibians are the group with the lowest number of studies (3.3%). Sensitivity to toxicants is species dependent (Malaj et al. 2012). However, sensitivity between different species is usually comparable when large sample sizes of species and taxonomic groups are used for toxicological models (Wang et al., 2014). In the case of invertebrates, this is a group with a high diversity of organisms. In the case of mollusks, they covered a wide range of sensitivities for metals (Malaj et al., 2012). For insects, Buchwalter and Luoma (2005) showed that variability of sensitivity within orders was larger than that between orders. Among vertebrates, the group of amphibians presented a few species in our models. However, Kerby et al. (2010) suggested that amphibians are not particularly sensitive to most contaminants. Our review has used a total of 729 species for the models from 1,318 studies with a wide representation of taxonomic groups (both vertebrates and invertebrates). Two groups with a wide representation and that also present a high sensitivity for most toxicants were crustaceans (204 species, 28% of the total number of species) and fish (171 species, 23.5% of the total number of species). Both groups are widely used for studies of reproduction, growth, and behavior (Agatz et al., 2013; Alonso et al., 2009; Biesinger et al., 2002; Bryan et al., 1995; Hellou, 2011; Martins et al., 2017; Sadeq and Beckerman, 2019), including both, short- and long-term studies. Therefore, our models are a good representation of the taxonomic variability, endpoints, and range of concentrations that are studied in ecotoxicology.

Although previous studies have shown that reproduction bioassays need longer exposures than behavioral studies (see Melvin and Wilson, 2013 for review), in our study the Screening Point Value for Time (SPVT5) for reproduction was similar to SPVT5 for behavior (0.013 vs 0.012 days). This was an unexpected result. However, our reproduction models included reproduction studies with short-term exposures (less than 1 day). Those studies tested the effects of metals-metalloids on the sperm fitness (e. g. mussel sperm, Fitzpatrick et al., 2008) or egg fertilization (e. g. polychaete eggs, Gopalakrishnan et al. 2008). Both parameters cause clear effects on reproduction fitness at long-term. When sperm of polychaetes were exposed to different metals, the rate of fertilization decreased (Gopalakrishnan et al. 2008). The exposure of sperm to copper in mussels caused a reduction in its swimming speed, which may cause a deleterious effect on reproduction (Fitzpatrick et al., 2008). However, most of the studies used in our reproduction model are conducted with exposure-times longer than one week, reproduction studies of 21 days with cladocerans being especially frequent, as this is the time of the standardized reproduction bioassays with this invertebrate group (Biesinger et al., 2002; OECD 2012).

We can conclude that, for metal-metalloid toxicity, behavioral bioassays are less time-consuming than growth bioassays. However, reproduction bioassays present a similar exposure duration as behavioral studies. Reproduction and growth are endpoints that use less toxicant concentration by time unit than behavioral endpoints. In general, the sensitivity of behavioral studies was similar to that of growth and reproduction studies, suggesting that behavior may be used as an early warning signal of exposure to toxicant. This is especially useful with feeding behavior, as deleterious effects on feeding are related to growth and reproduction impairments. Both parameters are closely related to population dynamics, so behavior could be a promising link with population, community, and ecosystem organization levels. This study also highlights the effectiveness of short-term behavioral bioassays for assessing the exposure of aquatic organisms to chemical compounds.
